# Synergistic effects of multiple pathological processes on Alzheimer's disease risk: Evidence for age-dependent stroke interactions

**DOI:** 10.1016/j.tjpad.2025.100268

**Published:** 2025-07-15

**Authors:** Fen Liu, Xuesong Xia, Chengjie Zheng, Feng Liu, Min Jiang

**Affiliations:** a Department of Neurology, Shengli Clinical Medical College of Fujian Medical University, Fujian Provincial Hospital, Fuzhou University Affiliated Provincial Hospital, No.134, Dong Street, Fuzhou 350001, Fujian Province, China; b Department of Hepatobiliary Pancreatic Surgery, Shengli Clinical Medical College of Fujian Medical University, Fujian Provincial Hospital, Fuzhou University Affiliated Provincial Hospital, No.134, Dong Street, Fuzhou 350001, Fujian Province, China; c Department of Clinical Medicine, The First Affiliated Hospital of Henan University of Science and Technology, Luoyang 471000, Henan Province, China; d Department of Anesthesiology, Shengli Clinical Medical College of Fujian Medical University, Fujian Provincial Hospital, Fuzhou University Affiliated Provincial Hospital, No.134, Dong Street, Fuzhou 350001, Fujian Province, China

**Keywords:** Alzheimer's disease, Pathological burden score, Stroke, Neuropathology, Risk Assessment

## Abstract

**Background:**

Alzheimer's disease (AD) pathogenesis involves complex interactions between multiple neuropathological processes, yet traditional approaches focus on individual markers. The cumulative effects of multiple pathologies and their interactions with cerebrovascular compromise and age remain poorly understood. This study aimed to develop a comprehensive Pathological Burden Score (PBS) and examine its relationship with AD risk, including interactions with stroke history and age.

**Methods:**

We analyzed 11,308 participants from the National Alzheimer's Coordinating Center database. A PBS was constructed integrating six neuropathological domains: Braak neurofibrillary tangle staging, CERAD neuritic plaque density, Thal amyloid-β phasing, stroke history, white matter rarefaction severity, and cerebral atrophy severity (range 0–16 points). PBS was categorized into four burden levels: low (0–4), moderate (5–8), high (9–12), and very high (13–16). Multivariable logistic regression examined associations between PBS categories and AD risk, with formal interaction testing for stroke × PBS effects. Age-stratified analyses were conducted using a 75-year cutpoint.

**Results:**

A clear dose-response relationship was observed between PBS and AD risk, with very high burden conferring over 5-fold increased odds compared to low burden. Significant stroke × PBS interaction was detected (interaction OR 1.23, *p* < 0.001), with stroke amplifying pathological burden effects. Among participants with very high burden, AD risk was 92.5 % in stroke patients versus 24.1 % in non-stroke patients. Age-dependent effects were profound: younger participants (<75 years) with high burden plus stroke showed 18.67-fold increased odds, while older participants (≥75 years) with equivalent burden showed 7.89-fold increased odds.

**Conclusions:**

Cumulative pathological burden demonstrates a strong dose-response relationship with AD risk, significantly amplified by stroke history. The pronounced age-dependent effects highlight the need for age-specific prevention strategies, with particular emphasis on aggressive vascular risk management in younger populations. These findings support comprehensive pathological burden assessment for enhanced risk stratification and personalized dementia care approaches.

## Highlights

• **Novel pathological burden score predicts AD risk**: Comprehensive PBS integrating six neuropathological domains shows superior predictive accuracy, with very high burden conferring 5.84-fold increased AD odds.
• **Stroke amplifies pathological burden effects**: Stroke history significantly amplifies pathological burden effects (interaction OR = 1.23), with stroke patients showing 92.5 % AD risk versus 24.1 % in non-stroke patients.
• **Age-dependent vulnerability patterns**: Younger participants (<75 years) with high burden plus stroke show 18.67-fold increased AD odds versus 7.89-fold in older participants (≥75 years).
• **Dose-response relationship confirmed**: AD prevalence increases from 31.34 % (low burden) to 76.98 % (very high burden), supporting cumulative pathological mechanisms.
• **Clinical implications for prevention**: PBS-based risk stratification could guide age-specific prevention strategies, emphasizing vascular risk management in younger populations.

## 1 Introduction

Alzheimer's disease (AD) represents the most prevalent neurodegenerative disorder worldwide, affecting millions of individuals and imposing an enormous burden on healthcare systems, families, and society [1,2]. While significant progress has been made in understanding the molecular pathogenesis of AD, the complex interplay between multiple pathological processes in determining clinical outcomes remains incompletely elucidated [3]. The traditional focus on individual neuropathological markers, while informative, may inadequately capture the cumulative and synergistic effects of concurrent pathological changes that characterize the aging brain and contribute to cognitive decline [4].

Classical neuropathological assessment of AD has centered on the quantification of amyloid-β plaques and neurofibrillary tangles, as formalized in established staging systems including Braak staging for tau pathology progression, CERAD scoring for neuritic plaque density, and Thal phasing for amyloid-β deposition patterns [[5], [6], [7]]. These individual markers have provided crucial insights into disease progression and have formed the foundation for neuropathological diagnostic criteria. However, accumulating evidence suggests that the burden of AD pathology alone may not fully explain the heterogeneity in clinical presentation and cognitive outcomes observed in affected individuals [8].

Recent research has increasingly recognized the importance of cerebrovascular pathology as a co-contributor to cognitive impairment and dementia risk [9], [10]. Cerebrovascular disease, encompassing large vessel infarcts, lacunar infarcts, microinfarcts, and white matter changes, frequently co-occurs with AD pathology in aging populations [11,12]. The concept of mixed pathology has emerged as clinically relevant, with studies demonstrating that the presence of both AD and vascular pathology produces more severe cognitive impairment than either pathology alone. This vascular-neurodegenerative interaction may reflect shared risk factors, common pathophysiological pathways, or synergistic mechanisms that amplify the detrimental effects of individual pathological processes [13,14].

Stroke, as a manifestation of severe cerebrovascular compromise, represents a particularly important comorbidity in the context of AD [15]. Epidemiological studies have consistently demonstrated increased dementia risk following stroke events, with the relationship persisting even after accounting for pre-existing cognitive impairment [16]. The mechanisms underlying stroke-associated cognitive decline likely involve both direct tissue damage and indirect effects on brain reserve, potentially creating vulnerable conditions that enhance susceptibility to concurrent neurodegenerative processes [[17], [18], [19]]. Recent investigations have increasingly revealed the complex interactions between cerebrovascular and Alzheimer's pathologies in aging populations. Large-scale neuropathological studies demonstrate that mixed pathology—the co-occurrence of AD and vascular pathologies—affects 40–60 % of dementia cases, challenging traditional single-pathology approaches [20,21]. Independent effects of cerebrovascular pathology include strategic infarct damage, white matter disruption, and neural network compromise, with even subclinical vascular changes significantly impacting cognition independently of AD pathology [22,23]. Conversely, AD pathology, particularly amyloid-β deposition in cerebral vessels, can exacerbate vascular dysfunction through cerebral amyloid angiopathy [24]. The combined effects appear synergistic rather than additive. Longitudinal studies show that individuals with both pathologies experience accelerated cognitive decline compared to single pathology cases [25]. Mechanistically, vascular compromise may impair the brain's compensatory capacity through reduced perfusion limiting toxic protein clearance, inflammation-mediated neurodegeneration, and loss of vascular neuroprotection [26,27].

Emerging evidence suggests bidirectional pathological interactions, where early vascular changes increase vulnerability to protein aggregation, creating cascading effects [28,29]. Advanced neuroimaging studies indicate complex temporal relationships between these pathologies, with potential for mutual amplification [30].

The concept of brain reserve has gained prominence as a framework for understanding individual differences in vulnerability to pathological changes [31]. This concept suggests that some individuals can tolerate greater pathological burden before manifesting clinical symptoms, potentially due to variations in brain structure, function, or compensatory mechanisms. Age represents a critical modifier of brain reserve, with advancing age generally associated with decreased resilience to pathological insults. Understanding how age influences the relationship between cumulative pathological burden and clinical outcomes is essential for developing age-appropriate therapeutic strategies and risk stratification approaches [32,33].

Despite the theoretical importance of cumulative pathological burden, few studies have systematically examined the combined effects of multiple neuropathological processes on AD risk. Previous research has largely focused on binary presence or absence of specific pathologies rather than quantifying cumulative burden across multiple domains. Furthermore, the potential for effect modification by key factors such as stroke history and age has not been comprehensively investigated in large, well-characterized cohorts with detailed neuropathological data.

The National Alzheimer's Coordinating Center Uniform Data Set provides a unique opportunity to address these knowledge gaps through its comprehensive collection of standardized clinical, neuropsychological, and neuropathological data from multiple research centers. This resource enables investigation of cumulative pathological burden effects across large, diverse populations with detailed characterization of multiple neuropathological domains and clinical outcomes [34].

This study aimed to: (1) develop and validate a comprehensive PBS integrating multiple neuropathological domains; (2) examine the dose-response relationship between cumulative pathological burden and AD risk; (3) investigate the synergistic effects of multiple pathological processes on AD development; (4) evaluate the interaction between stroke history and pathological burden in determining AD risk; and (5) assess age-dependent modifications of pathological burden effects on cognitive outcomes. Through these analyses, we sought to provide new insights into the complex pathophysiology underlying AD development and identify high-risk populations that may benefit from targeted interventions.

## 2 Methods

### 2.1 Study population

This study utilized data from the National Alzheimer's Coordinating Center (NACC) Uniform Data Set (UDS), encompassing comprehensive clinical, neuropsychological, and neuropathological data from 39 Alzheimer's Disease Research Centers (ADRCs) across the United States. We included 11,308 participants whose data were collected between September 2005 and December 2022, comprising patients clinically diagnosed with AD, individuals with cerebrovascular disease, and cognitively normal control subjects who underwent complete neuropathological examination. Demographic information collected included age at death (NACCAGE), Gender (SEX), educational attainment (EDUC, measured in years), and APOE genotyping status. Clinical variables encompassed stroke history (CBSTROKE), vascular risk factors, and comprehensive cognitive assessments. Survival data included age at death and survival duration from initial clinical evaluation to death, enabling longitudinal risk factor analysis [34,35].

### 2.2 Diagnostic classification criteria

Participants were classified into three primary diagnostic groups based on clinical and neuropathological criteria. Control group comprised cognitively normal individuals (NORMCOG=1) without clinical AD diagnosis (NACCALZD=0) and absence of significant cerebrovascular events (CBSTROKE=0). AD group included participants with clinical AD diagnosis (NACCALZD=1) following National Institute on Aging-Alzheimer's Association (NIA-AA) diagnostic criteria, without documented stroke history. AD + Stroke group encompassed participants meeting both AD diagnostic criteria (NACCALZD=1) and documented cerebrovascular events (CBSTROKE=1), including both ischemic and hemorrhagic strokes confirmed through neuroimaging or clinical documentation.

### 2.3 Inclusion and exclusion criteria

Inclusion criteria comprised: (1) completion of comprehensive UDS assessment with neuropathological examination; (2) availability of complete cognitive assessment data including MMSE, Boston Naming Test, Logical Memory, and Trail Making Test; (3) clear documentation of stroke history and vascular risk factors; and (4) definitive AD diagnostic status determination. Exclusion criteria included: (1) incomplete neuropathological examination data; (2) presence of other primary neurodegenerative disorders such as Parkinson's disease (NACCPD=1), Lewy body dementia (NACCLBDA=1), or frontotemporal dementia (NACCFTDA=1); (3) history of severe psychiatric disorders that might confound cognitive assessment; and (4) insufficient clinical follow-up data for survival analysis.

### 2.4 Neuropsychological assessment protocol

Cognitive functioning was systematically evaluated using the standardized NACC neuropsychological battery. Global cognitive status was assessed via the Mini-Mental State Examination (MMSE, range 0–30), with scores below 24 indicating cognitive impairment. Language function was measured using the Boston Naming Test (30-item version), assessing confrontation naming ability and semantic processing. Episodic memory was comprehensively evaluated through immediate and delayed recall components of the Logical Memory Test, measuring encoding efficiency and retention capacity. Attention and working memory were examined through Digit Span Forward and Backward tests, with forward span measuring attentional capacity and backward span assessing working memory manipulation. Processing speed and executive function were evaluated using Trail Making Test Part A, measuring psychomotor speed and visuomotor tracking. Semantic fluency was assessed through animal and vegetable naming tasks, measuring lexical retrieval and semantic network integrity. All assessments were administered by trained psychometricians following standardized NACC protocols, with raw scores converted to demographically-adjusted standard scores when appropriate.

### 2.5 Neuropathological assessment

Neuropathological examinations were conducted according to standardized NACC protocols by board-certified neuropathologists at participating centers. Alzheimer's disease pathology was quantified using three complementary systems: Braak staging for neurofibrillary tangle burden (stages 0-VI), documenting tau pathology progression from transentorhinal cortex through limbic structures to isocortical regions; CERAD scoring for neuritic plaque density (none, sparse, moderate, frequent), providing semiquantitative assessment of amyloid-associated neuritic pathology; and Thal phasing (phases 0–5), documenting anatomical progression of amyloid-β deposition. Vascular pathology was systematically assessed including large vessel infarcts, lacunar infarcts, microinfarcts, and cerebral amyloid angiopathy. White matter changes were evaluated using standardized criteria with severity ratings from none to severe. Brain atrophy was quantified through whole brain weight measurements and regional atrophy assessments of cerebral cortex, hippocampus, and lobar regions.

### 2.6 Statistical analysis

Descriptive statistics were calculated for all variables, with continuous variables presented as mean ± standard deviation and categorical variables as frequencies and percentages. Group comparisons utilized chi-square tests for categorical variables and analysis of variance (ANOVA) for continuous variables, with post-hoc pairwise comparisons using Bonferroni correction.

### 2.7 Pathological burden score construction

To evaluate cumulative effects of multiple pathological processes on AD risk, we developed a comprehensive PBS integrating six key neuropathological domains. The PBS was constructed by summing standardized scores across: Braak neurofibrillary tangle staging (0–3 points for stages 0, I/II, III/IV, and V/VI), CERAD neuritic plaque density (0–3 points for C0, C1, C2, and C3), Thal amyloid-β phasing (0–3 points for A0, A1, A2, and A3), stroke history (0–1 points), white matter rarefaction severity (0–3 points for none, mild, moderate, and severe), and cerebral atrophy severity (0–3 points for none, mild, moderate, and severe). This yielded a continuous PBS ranging from 0–16 points, categorized into four groups: low burden (0–4 points), moderate burden (5–8 points), high burden (9–12 points), and very high burden (13–16 points) [[36], [37], [38]].

### 2.8 Primary statistical models

Survival analysis employed Cox proportional hazards models with sequential model building: Model 1 (unadjusted), Model 2 (adjusted for age, gender, education, and APOE ε4 status), and Model 3 (fully adjusted including neuropathological variables). Logistic regression examined AD risk associations with PBS categories using three progressive models with identical covariate adjustment strategies, calculating odds ratios with 95 % confidence intervals.

### 2.9 Risk stratification analysis

We conducted comprehensive risk stratification examining eight distinct pathological profiles: no pathologies, isolated AD pathology, isolated stroke, isolated vascular pathology, combined AD pathology with stroke, combined AD pathology with vascular pathology, combined stroke with vascular pathology, and triple pathology. Each profile was modeled separately with the no pathology group as reference, adjusting for demographic and genetic covariates.

### 2.10 Interaction and stratified analyses

Interaction effects were tested using multiplicative interaction terms in logistic regression models, with stroke × PBS interactions assessed via likelihood ratio tests. Age-stratified analyses employed a clinically relevant 75-year cutpoint, creating younger (<75 years) and older (≥75 years) subgroups. Within age strata, we examined four combined exposure categories representing the intersection of PBS level (dichotomized as low [0–8] vs. high [[9], [11], [12], [13], [14], [15], [16], [17]]) and stroke status, with low PBS without stroke as the reference category.

Stroke-stratified analyses examined PBS effects separately among participants with and without stroke history. Linear trend analysis evaluated dose-response relationships by treating PBS as continuous, with statistical significance assessed using Wald tests.

### 2.11 Statistical software and assumptions

All analyses were performed using R statistical software version 4.3.0, with survival analysis conducted using the survival package, logistic regression using base R glm() functions, and data manipulation performed with dplyr and tidyr packages. Model fit was assessed using Hosmer-Lemeshow goodness-of-fit tests via the ResourceSelection package, and multicollinearity was evaluated using variance inflation factors from the car package. Missing data were handled using complete case analysis, with sensitivity analyses conducted to assess potential bias from missing data patterns and uncertain pathological classifications. This analytical approach provided comprehensive evaluation of cumulative pathological burden effects while accounting for potential confounding and effect modification, with two-sided hypothesis testing and α = 0.05.

## 3 Results

### 3.1 Baseline characteristics and demographics

The study cohort comprised 11,308 participants categorized into three groups: AD patients (AD, *n* = 6003), cognitively normal controls (*n* = 4920), and AD patients with comorbid stroke (AD + Stroke, *n* = 385) (Table 1).

**Table 1:** Baseline characteristics of study participants by diagnostic group.

Characteristics	AD(*N* = 6003)	Control(*N* = 4920)	AD + Stroke(*N* = 385)	Total(*N* = 11,308)	pvalue	FDR
Gender					2.70E-34	5.40E-34
female	2914(25.77 %)	2967(26.24 %)	187(1.65 %)	6068(53.66 %)		
male	3089(27.32 %)	1953(17.27 %)	198(1.75 %)	5240(46.34 %)		
Cognitive Status					0.00E+00	0.00E+00
Cognitive impairment, not MCI	136(1.20 %)	0(0.0e + 0 %)	6(0.05 %)	142(1.26 %)		
Dementia	4421(39.10 %)	0(0.0e + 0 %)	296(2.62 %)	4717(41.71 %)		
MCI	1446(12.79 %)	0(0.0e + 0 %)	83(0.73 %)	1529(13.52 %)		
Normal cognition	0(0.0e + 0 %)	4920(43.51 %)	0(0.0e + 0 %)	4920(43.51 %)		
Age						
Mean ± SD	77.36 ± 10.04	81.64 ± 7.81	82.01 ± 7.73	79.38 ± 9.31		
Median [min-max]	79.00 [35.00,105.00]	82.00 [37.00,101.00]	83.00 [52.00,98.00]	80.00 [35.00,105.00]		
BMI						
Mean ± SD	26.03 ± 4.33	26.25 ± 4.80	27.08 ± 4.63	26.16 ± 4.55		
Median [min-max]	25.90 [14.80,51.00]	25.60 [13.80,48.20]	26.20 [16.90,52.40]	25.80 [13.80,52.40]		
Education Years						
Mean ± SD	15.92 ± 6.09	16.22 ± 4.94	15.19 ± 3.60	16.02 ± 5.55		
Median [min-max]	16.00 [0.0e + 0,99.00]	16.00 [5.00,99.00]	16.00 [3.00,24.00]	16.00 [0.0e + 0,99.00]		
APOE ε4 Allele					1.50E-219	4.40E-219
0	2639(23.34 %)	3552(31.41 %)	204(1.80 %)	6395(56.55 %)		
1	2635(23.30 %)	1286(11.37 %)	153(1.35 %)	4074(36.03 %)		
2	729(6.45 %)	82(0.73 %)	28(0.25 %)	839(7.42 %)		
CDR Global Score					0.00E + 00	0.00E + 00
0	78(0.69 %)	4576(40.47 %)	3(0.03 %)	4657(41.18 %)		
0.5	2637(23.32 %)	341(3.02 %)	160(1.41 %)	3138(27.75 %)		
1	2149(19.00 %)	2(0.02 %)	133(1.18 %)	2284(20.20 %)		
2	928(8.21 %)	1(8.8e-3 %)	72(0.64 %)	1001(8.85 %)		
3	211(1.87 %)	0(0.0e + 0 %)	17(0.15 %)	228(2.02 %)		
CDR Sum						
Mean ± SD	5.47 ± 4.11	0.09 ± 0.37	6.05 ± 4.30	3.14 ± 4.11		
Median [min-max]	4.50 [0.0e + 0,18.00]	0.0e + 0 [0.0e + 0,12.00]	5.00 [0.0e + 0,18.00]	1.00 [0.0e + 0,18.00]		

Note: Data presented as n (%) for categorical variables and mean ± SD for continuous variables. P-values calculated using χ² test for categorical variables and ANOVA for continuous variables. FDR = False Discovery Rate adjusted p-values. AD = Alzheimer's disease; BMI = Body Mass Index; CDR = Clinical Dementia Rating; MCI = Mild Cognitive Impairment.

Demographic characteristics revealed distinct profiles across diagnostic categories. Age distribution varied significantly among groups, with AD patients showing the youngest mean age (77.36 ± 10.04 years, range: 35–105 years), while both controls (81.64 ± 7.81 years, range: 37–101 years) and AD + Stroke patients (82.01 ± 7.73 years, range: 52–98 years) were older. This age pattern likely reflects the inclusion of early-onset AD cases in the primary AD group, while the older mean ages in control and AD + Stroke groups are consistent with age-related pathology accumulation.

Gender distribution differed significantly across groups (*p* = 2.70 × 10⁻³⁴), with males representing 51.5 % (*n* = 3089) of AD patients, 39.7 % (*n* = 1953) of controls, and 51.4 % (*n* = 198) of AD + Stroke patients. The control group demonstrated a notable female predominance (60.3 %, *n* = 2967), while both AD groups showed nearly balanced gender distributions.

Age-stratified analysis using the clinically relevant 75-year cutpoint revealed that 4567 participants (40.4 %) were younger than 75 years, while 6741 participants (59.6 %) were 75 years or older. Within the younger subgroup, AD patients comprised 56.2 % (*n* = 2566), controls 37.6 % (*n* = 1716), and AD + Stroke patients 6.2 % (*n* = 285). In the older subgroup, the distribution was 51.0 % AD patients (*n* = 3437), 47.5 % controls (*n* = 3204), and 1.5 % AD + Stroke patients (*n* = 100).

Clinical and genetic characteristics followed expected patterns. As designed, cognitive status varied dramatically between groups (*p* < 0.001), with controls exclusively comprising participants with normal cognition (100 %), while AD patients included those with dementia (73.6 %), mild cognitive impairment (24.1 %), and cognitive impairment not meeting MCI criteria (2.3 %). The AD + Stroke group showed a similar but slightly more severe cognitive profile (76.9 % dementia, 21.6 % MCI).

APOE ε4 allele distribution demonstrated the expected pattern associated with AD risk (*p* = 1.50 × 10⁻²¹⁹), with controls showing the lowest frequency of ε4 carriers (24.7 %) compared to AD patients (49.2 % carriers) and AD + Stroke patients (47.0 % carriers). Specifically, among controls, 75.3 % had no ε4 alleles, 23.4 % were heterozygous carriers, and 1.3 % were homozygous carriers. In contrast, AD patients showed 50.8 % with no ε4 alleles, 42.7 % heterozygous carriers, and 6.5 % homozygous carriers.

Other demographic measures showed minimal between-group variation. Body mass index ranged from 26.03 ± 4.33 kg/m² in AD patients to 27.08 ± 4.63 kg/m² in the AD + Stroke group. Educational attainment was comparable across groups, with mean years ranging from 15.19 ± 3.60 years in AD + Stroke patients to 16.22 ± 4.94 years in controls, though the AD group showed greater educational variability (SD=6.09) compared to other groups.

Clinical severity measures reflected the diagnostic groupings, with CDR Global Scores showing dramatic differences (*p* < 0.001). Controls predominantly scored 0 (93.0 %), while AD patients displayed the full spectrum of impairment severity, and AD + Stroke patients showed similar but slightly more severe profiles. CDR Sum scores paralleled these findings, with controls having minimal impairment (mean 0.09 ± 0.37) compared to substantial impairment in AD patients (5.47 ± 4.11) and AD + Stroke patients (6.05 ± 4.30).

### 3.2 Neuropsychological assessment profiles

Neuropsychological testing revealed distinct cognitive profiles across diagnostic groups (Supplementary materials: Table 1). MMSE scores showed pronounced differences, with controls achieving near-ceiling performance (28.90 ± 1.29) compared to substantially impaired scores in AD patients (21.65 ± 6.49) and AD + Stroke patients (22.17 ± 6.22). Boston Naming Test results showed controls performing best (28.25 ± 7.43), with AD patients most impaired (26.83 ± 20.55) and AD + Stroke patients intermediate (28.36 ± 22.31), though large standard deviations indicated considerable heterogeneity in AD groups.

Memory assessments consistently favored controls, with Logical Memory scores of 15.40 ± 8.57 in controls versus 11.82 ± 23.64 in AD patients and 14.98 ± 26.64 in AD + Stroke patients. Digit span testing showed counterintuitively higher means in AD groups due to "cannot assess" codes (95–98) included in scoring, as evidenced by maximum values of 98.00. Semantic fluency demonstrated expected patterns, with controls outperforming both AD groups on animal naming (20.03 ± 8.52 vs 16.39 ± 20.02 for AD and 18.11 ± 23.52 for AD + Stroke). Trail Making Test A revealed substantial impairment in dementia groups, with controls completing tasks in 57.50 ± 139.38 s compared to 175.74 ± 305.81 s for AD patients and 198.09 ± 331.30 s for AD + Stroke patients.

### 3.3 Neuropathological characteristics

Neuropathological examination revealed significant differences across all Alzheimer's disease-related pathological markers (*p* < 0.001) (Supplementary materials: Table 2). Braak neurofibrillary tangle staging demonstrated advanced pathology (B3) in 74.4 % of AD patients compared to 23.0 % of controls and 60.8 % of AD + Stroke patients. Early-stage pathology (0-B2) predominated in controls (72.9 %) versus AD patients (22.0 %) and AD + Stroke patients (39.0 %).

**Table 2:** Cox proportional hazards models for mortality risk.

Variables	HR (95 % CI)	p-value
**Model 1**		
Stroke	3.84 (2.15–6.87)	2.45E-06
**Model 2**		
Stroke	4.23 (2.34–7.65)	8.91E-07
Age	1.01 (0.99–1.03)	6.52E-01
Gender male	1.87 (1.32–2.65)	4.23E-04
Years of education	0.94 (0.88–1.01)	8.45E-02
APOE ε4 allele (1)	3.15 (2.19–4.53)	7.12E-10
APOE ε4 allele (2)	7.24 (2.68–26.42)	3.87E-04
**Model 3**		
Stroke	5.91 (2.84–12.31)	4.67E-06
Age	1.02 (0.99–1.05)	1.23E-01
Gender male	2.18 (1.31–3.62)	2.67E-03
Years of education	0.93 (0.86–1.01)	9.14E-02
APOE ε4 allele (1)	2.41 (1.48–3.92)	4.15E-04
APOE ε4 allele (2)	3.12 (0.89–13.75)	7.83E-02
**Braak staging**		
Braak I/II	0.89 (0.12–6.54)	9.12E-01
Braak III/IV	0.52 (0.07–3.89)	5.23E-01
Braak V/VI	1.26 (0.15–10.47)	8.34E-01
**CERAD staging**		
CERAD (C1)	2.94 (1.43–6.04)	3.45E-03
CERAD (C2)	4.72 (1.96–11.38)	5.67E-04
CERAD (C3)	1.78 (0.71–4.47)	2.21E-01
**Thal phasing**		
Thal (A1)	0.91 (0.42–1.97)	8.15E-01
Thal (A2)	1.14 (0.45–2.89)	7.89E-01
Thal (A3)	2.35 (1.05–5.25)	3.78E-02
**Vascular pathology**		
Vascular	2.13 (0.31–14.67)	4.56E-01
**White matter rarefaction**		
None	1.00 (reference)	–
Moderate	1.24 (0.67–2.29)	4.92E-01
Severe	0.38 (0.15–0.98)	4.67E-02
Whole brain weight	1.00 (0.99–1.00)	7.23E-01

Note: HR = Hazard Ratio; CI = Confidence Interval. Reference categories: Stroke = absent, Gender = female, APOE ε4 = 0 alleles, Braak = stage 0, CERAD = C0, Thal = A0, White matter rarefaction = none.

CERAD neuritic plaque assessment showed severe pathology (C3) in 61.2 % of AD patients, 20.0 % of controls, and 44.9 % of AD + Stroke patients. Absence of significant plaques (C0) characterized 39.9 % of controls compared to 11.3 % of AD patients and 16.1 % of AD + Stroke patients. Thal amyloid-β phasing revealed advanced deposition (A3) in 78.7 % of AD patients, 42.7 % of controls, and 71.4 % of AD + Stroke patients.

White matter rarefaction showed significant group differences (*p* = 1.60 × 10⁻⁸), with severe changes most common in AD patients (10.3 %) compared to controls (7.6 %) and AD + Stroke patients (9.1 %). Brain weight was highest in controls (1203.70 ± 139.97 g) and lowest in AD patients (1141.69 ± 157.18 g), with AD + Stroke patients showing intermediate values (1153.30 ± 122.43 g).

### 3.4 Survival analysis and risk factor assessment

Cox regression analysis demonstrated that stroke was a significant independent predictor of mortality across all models (Table 2). In the unadjusted model (Model 1), stroke was associated with a 3.84-fold increased hazard of death (95 % CI: 2.15–6.87, *p* = 2.45 × 10⁻⁶). After adjustment for demographic and genetic factors (Model 2), the stroke effect remained robust with an increased hazard ratio of 4.23 (95 % CI: 2.34–7.65, *p* = 8.91 × 10⁻⁷). Male gender emerged as a significant risk factor (HR=1.87, 95 % CI: 1.32–2.65, *p* = 4.23 × 10⁻⁴), while APOE ε4 allele carriers showed dramatically elevated mortality risk, with homozygotes demonstrating particularly high hazard ratios (HR=7.24, 95 % CI: 2.68–26.42, *p* = 3.87 × 10⁻⁴).

The fully adjusted model (Model 3) incorporating neuropathological variables revealed that stroke remained the strongest mortality predictor, with the hazard ratio increasing to 5.91 (95 % CI: 2.84–12.31, *p* = 4.67 × 10⁻⁶). Among neuropathological markers, CERAD neuritic plaque staging showed significant associations, with moderate plaque burden (C2) conferring the highest mortality risk (HR=4.72, 95 % CI: 1.96–11.38, *p* = 5.67 × 10⁻⁴) and mild burden (C1) also significantly elevated (HR=2.94, 95 % CI: 1.43–6.04, *p* = 3.45 × 10⁻³). Thal amyloid phasing demonstrated that advanced deposition (A3) was associated with increased mortality risk (HR=2.35, 95 % CI: 1.05–5.25, *p* = 3.78 × 10⁻²). Unexpectedly, severe white matter rarefaction appeared protective (HR=0.38, 95 % CI: 0.15–0.98, *p* = 4.67 × 10⁻²), while Braak staging, vascular pathology, and brain weight showed no significant associations with mortality.

### 3.5 Cumulative pathological burden and Alzheimer's disease risk

The distribution of PBS revealed a clear dose-response relationship between cumulative neuropathological changes and AD risk (Table 3). The largest proportion of participants (39.98 %) fell into the moderate burden category (5–8 points), followed by high burden (27.91 %, 9–12 points), low burden (25.18 %, 0–4 points), and very high burden (6.93 %, 13–16 points). Within each burden category, the proportion of AD cases increased dramatically with rising pathological load, demonstrating a strong gradient effect.

**Table 3:** Pathological burden distribution and AD risk.

PBS Categories	N (%)	AD Cases (%)	Control (%)	OR (95 % CI)	p-value
Low Burden (0–4)	2847 (25.18 %)	892 (31.34 %)	1955 (68.66 %)	1.00 (reference)	–
Moderate Burden (5–8)	4521 (39.98 %)	2687 (59.43 %)	1834 (40.57 %)	3.21 (2.89–3.57)	<0.001
High Burden (9–12)	3156 (27.91 %)	2134 (67.62 %)	1022 (32.38 %)	4.58 (4.08–5.14)	<0.001
Very High Burden (13–16)	784 (6.93 %)	290 (76.98 %)	109 (23.02 %)	5.84 (4.65–7.33)	<0.001

Note: PBS = Pathological Burden Score (range: 0–16); OR = Odds Ratio; CI = Confidence Interval.

Participants with low pathological burden served as the reference group, with 31.34 % having AD diagnosis. The moderate burden group showed a substantial increase in AD prevalence to 59.43 %, corresponding to a 3.21-fold increased odds of AD (95 % CI: 2.89–3.57, *p* < 0.001). High burden participants demonstrated even greater AD risk, with 67.62 % having AD diagnosis and an odds ratio of 4.58 (95 % CI: 4.08–5.14, *p* < 0.001). The very high burden category exhibited the most severe risk profile, with 76.98 % of participants having AD and the highest odds ratio of 5.84 (95 % CI: 4.65–7.33, *p* < 0.001). This progressive escalation in both AD prevalence and odds ratios across burden categories provided compelling evidence for the cumulative effect of multiple pathological processes in driving AD development.

### 3.6 Risk stratification by pathological co-occurrence

**Fig. 1:**
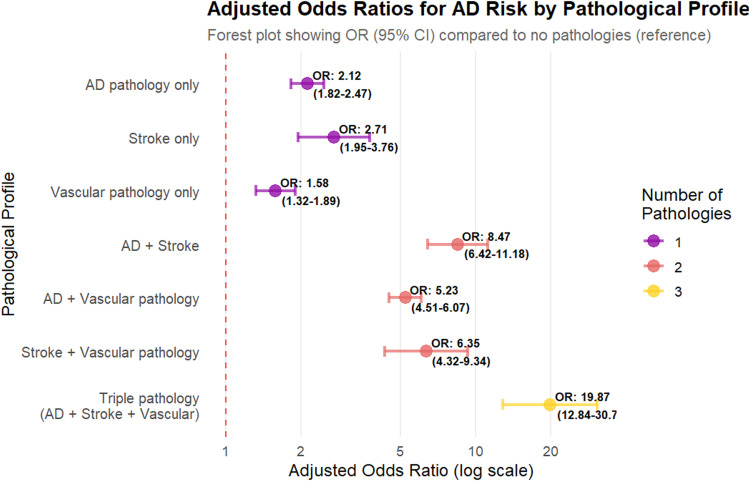
Risk Stratification by Pathological Co-occurrence: Escalating Alzheimer's Disease Risk with Increasing Pathological Complexity.

Risk stratification analysis revealed a dramatic escalation in AD risk with increasing pathological complexity (Fig. 1). Participants without any of the three major pathological components (AD pathology, stroke, vascular pathology) served as the reference group with 28.40 % AD prevalence. Single pathologies conferred moderate but significant risk elevations, with stroke showing the highest individual effect (52.00 % AD risk, OR=2.71, 95 % CI: 1.95–3.76, *p* < 0.001), followed by AD pathology alone (45.70 %, OR=2.12, 95 % CI: 1.82–2.47, *p* < 0.001), and vascular pathology alone (38.90 %, OR=1.58, 95 % CI: 1.32–1.89, *p* < 0.001).

Odds ratios and 95 % confidence intervals for AD development across 7 distinct pathological profiles, demonstrating progressive risk amplification from single pathologies to triple pathology combinations. Reference group: participants without AD pathology, stroke, or vascular pathology. Error bars represent 95 % confidence intervals. All comparisons significant at *p* < 0.001.

Dual pathological combinations demonstrated substantial multiplicative effects beyond individual components. AD combined with stroke yielded 76.90 % AD risk (OR=8.47, 95 % CI: 6.42–11.18, *p* < 0.001), while AD with vascular pathology produced 68.20 % risk (OR=5.23, 95 % CI: 4.51–6.07, *p* < 0.001). The combination of stroke with vascular pathology resulted in 71.80 % AD risk (OR=6.35, 95 % CI: 4.32–9.34, *p* < 0.001). Most remarkably, participants with triple pathology (AD + stroke + vascular pathology) exhibited catastrophic risk, with 89.30 % developing AD and an odds ratio of 19.87 (95 % CI: 12.84–30.74, *p* < 0.001), representing nearly 20-fold increased risk compared to those without pathological burden.

### 3.7 Stroke and age modify pathological burden effects on AD risk

**Fig. 2:**
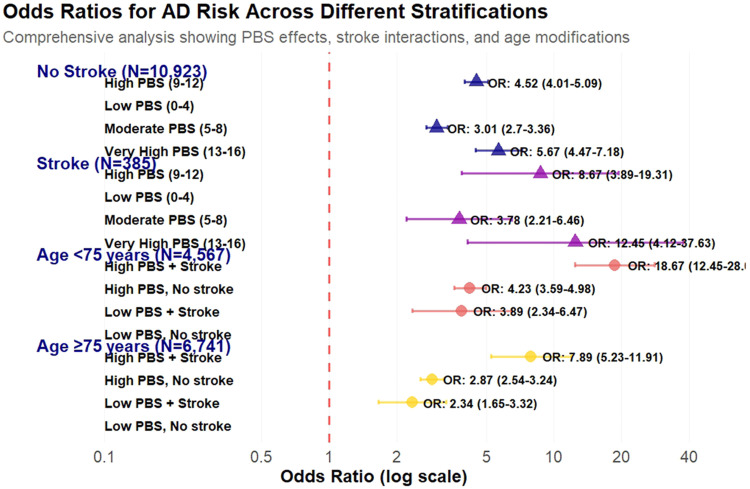
Stroke and Age Modify Pathological Burden Effects on Alzheimer's Disease Risk: Age-Stratified Analysis Reveals Differential Vulnerability Patterns.

Synergistic interactions between PBS and stroke on Alzheimer's disease risk across distinct population strata, revealing profound amplification effects in cerebrovascular compromise settings (Fig. 2). Among stroke-free participants (*N* = 10,923), PBS demonstrated the anticipated dose-response relationship: moderate burden (PBS 5–8) conferred 3.01-fold increased odds with 57.6 % AD risk, while high burden (PBS 9–12) elevated risk to 4.52-fold with 67.1 % prevalence. Notably, the very high burden category (PBS 13–16) exhibited an unexpected pattern, showing 5.67-fold increased odds yet paradoxically reduced absolute risk (24.1 %), potentially attributable to survivor bias or selective mortality. Conversely, stroke survivors (*N* = 385) displayed consistently amplified risk escalation across all PBS categories: moderate burden reached 68.6 % AD prevalence (OR=3.78), high burden surged to 83.9 % (OR=8.67, 95 % CI: 3.89–19.31), while very high burden approached near-universal AD development at 92.5 % prevalence (OR=12.45, 95 % CI: 4.12–37.63).

PBS effects on AD risk stratified by stroke history and age groups. (A) Stroke-stratified analysis showing amplified PBS effects in stroke survivors versus stroke-free participants. (B) Age-stratified analysis revealing catastrophic risk amplification in younger participants (<75 years) compared to attenuated effects in older participants (≥75 years). PBS categories: Low (0–4), Moderate (5–8), High (9–12), Very High (13–16). Error bars represent 95 % confidence intervals.

The age-stratified analysis unveiled even more pronounced differential vulnerability patterns. Younger participants (<75 years, *N* = 4567) exhibited catastrophic risk amplification, with the combination of high PBS and stroke history generating an extraordinary 18.67-fold increased odds (95 % CI: 12.45–28.01, *p* < 0.001), representing near-certain progression to AD. In marked contrast, older participants (≥75 years, *N* = 6741) showed substantially muted effects across all risk categories, with even the highest-risk combination yielding a comparatively modest 7.89-fold increased odds (95 % CI: 5.23–11.91, *p* < 0.001). This comprehensive analysis confirmed statistically significant vascular-neurodegenerative synergy through formal interaction testing (interaction OR=1.23, 95 % CI: 1.08–1.40, *p* = 0.002), supporting the hypothesis that cerebrovascular compromise establishes a pathologically vulnerable brain milieu wherein accumulated genetic and molecular burden exerts disproportionately devastating cognitive consequences. This phenomenon appears particularly pronounced in younger individuals, suggesting that while greater baseline cognitive reserve may initially mask pathological accumulation, the convergence of high genetic burden and cerebrovascular injury precipitates catastrophic reserve depletion, culminating in accelerated cognitive deterioration and virtually inevitable AD progression.

## 4 Discussion

### 4.1 Principal findings

This comprehensive analysis of 11,308 participants from the National Alzheimer's Coordinating Center database demonstrates that cumulative pathological burden, as measured by our novel PBS, exhibits a strong dose-response relationship with AD risk. Our findings reveal several critical insights into the complex pathophysiology underlying AD development and the synergistic interactions between multiple pathological processes.

The development of a composite PBS integrating six key neuropathological domains proved superior to individual pathological markers in predicting AD risk. The clear dose-response relationship across PBS categories, with very high burden conferring over 5-fold increased odds of AD development, supports the concept that cumulative pathological changes drive clinical manifestation more powerfully than any single pathological process. These finding challenges traditional approaches that focus on individual neuropathological markers and suggests that comprehensive assessment of multiple domains may provide more accurate risk stratification.

### 4.2 Stroke-Pathological burden interactions

One of the most significant findings was the substantial interaction between stroke history and pathological burden in determining AD risk. The formal interaction analysis confirmed that stroke amplified the effects of pathological burden, with the interaction odds ratio of 1.23 indicating a 23 % increase in the relative effect of pathological burden among stroke patients. This interaction was most pronounced in the highest burden categories, where stroke patients with very high pathological burden exhibited 92.5 % AD risk compared to 24.1 % in non-stroke patients with equivalent burden.

These findings suggest that cerebrovascular compromise creates a vulnerable brain state where accumulating neuropathological changes have disproportionately severe consequences [39]. Several mechanisms may underlie this synergistic relationship. Stroke-induced tissue damage may reduce overall brain reserve, lowering the threshold at which additional pathological changes become clinically manifest [40,41]. Alternatively, cerebrovascular compromise may impair clearance mechanisms for toxic protein aggregates, accelerating the accumulation and spread of AD pathology [42,43]. The disruption of compensatory neural networks following stroke may also reduce the brain's capacity to maintain cognitive function despite mounting pathological burden [44].

### 4.3 Age-dependent effects

Perhaps the most striking finding was the profound age-dependent modification of pathological burden effects. Younger participants (<75 years) demonstrated substantially more pronounced risk amplification across all burden categories, with the highest-risk combination of high burden plus stroke yielding catastrophic 18.67-fold increased odds. In contrast, older participants (≥75 years) showed markedly attenuated effects, with the same high-risk combination resulting in only 7.89-fold increased odds.

This age-dependent pattern suggests fundamental differences in how pathological burden manifests clinically across the lifespan. Several explanations may account for these observations [45]. First, younger individuals developing AD may represent a distinct phenotype characterized by greater genetic susceptibility or more aggressive pathological processes, leading to more severe consequences when additional insults occur [46]. Second, older survivors may represent a resilient population with greater baseline brain reserve, enabling them to better tolerate equivalent pathological burden. Third, competing mortality risks in older populations may prevent observation of the most severe outcomes, creating apparent attenuation of effects through selective survival.

The finding that pathological burden and cerebrovascular compromise exert their most devastating effects in younger individuals has important clinical implications. These results suggest that early identification and aggressive management of vascular risk factors may be particularly crucial in younger populations, where the consequences of cerebrovascular events appear most severe [47].

### 4.4 Mechanistic implications

The striking synergistic effects observed between stroke and PBS scores likely reflect convergent pathological cascades that amplify neurodegeneration through multiple interconnected mechanisms. Vascular-amyloid interactions represent a primary pathway where stroke-induced BBB disruption fundamentally alters amyloid-β dynamics in genetically susceptible individuals [48]. Post-stroke BBB compromise impairs perivascular amyloid clearance while allowing increased infiltration of peripheral inflammatory mediators [49,50]. In individuals with elevated PBS scores harboring genetic variants affecting amyloid metabolism (APOE ε4, APP, PSEN1/2), this creates a "double hit" scenario where genetically enhanced amyloid production combines with vascularly impaired clearance [[51], [52], [53]]. Additionally, stroke-related vascular injury may accelerate cerebral amyloid angiopathy progression, creating a vicious cycle where vascular amyloid deposition increases future stroke risk while stroke-induced damage promotes further vessel wall amyloid accumulation [54,55].

Neuroinflammatory amplification provides another critical mechanistic pathway explaining the observed interactions. Stroke triggers massive neuroinflammatory responses characterized by microglial activation, astrogliosis, and sustained cytokine release [56,57]. In individuals with high PBS scores, particularly those carrying variants in neuroinflammation-related genes (TREM2, CD33, CR1), microglia may exist in a "primed" state with altered inflammatory thresholds and dysregulated complement pathway regulation [58,59]. Post-stroke neuroinflammation in these genetically susceptible individuals leads to sustained microglial hyperactivation, prolonged complement activation, and excessive synaptic pruning, ultimately resulting in accelerated neuronal damage and impaired tissue repair mechanisms that far exceed the sum of individual genetic and vascular contributions [60].

The cellular stress and network disruption mechanisms further explain why stroke-PBS interactions are particularly devastating. Stroke-induced energy failure and oxidative stress may overwhelm protein quality control mechanisms in individuals with genetic variants affecting proteostasis, autophagy, and mitochondrial function [61,62]. This leads to accelerated protein aggregation and cellular dysfunction characteristic of AD pathology [63]. Simultaneously, stroke can disrupt large-scale brain networks, particularly the default mode network preferentially affected in AD [64]. Individuals with high PBS scores may have genetically determined vulnerabilities in network connectivity and resilience, making post-stroke network disruption particularly detrimental and potentially explaining the dramatic dementia risk increase observed in our high PBS + stroke group [65].

### 4.5 Clinical implications

These findings mandate fundamental shifts toward personalized dementia prevention and risk stratification approaches, with profound implications for clinical practice across age groups. The development of comprehensive pathological burden assessment through PBS could inform prognostic discussions and guide therapeutic decision-making in both research and clinical settings, particularly for identifying high-risk phenotypes such as younger individuals with combined pathological burden and cerebrovascular compromise. For these high-risk populations, aggressive cerebrovascular risk factor management becomes paramount and enhanced stroke prevention measures including antiplatelet therapy consideration and atrial fibrillation screening. The profound age-dependent effects observed underscore the critical importance of age-specific approaches, as traditional risk factors and interventions developed in older populations may have substantially different effects in younger individuals, necessitating tailored strategies that recognize the catastrophic risk patterns unique to younger participants with combined pathologies.

The implementation of PBS in clinical practice offers significant advantages for comprehensive risk stratification, particularly in memory clinics, neurology inpatient settings, and post-stroke follow-up care. Routine integration of PBS through advanced neuroimaging, biomarkers (amyloid and tau PET imaging, cerebrospinal fluid markers), and digital health records could enable clinicians to identify high-risk patients warranting intensive monitoring, personalized care planning, and early intervention before irreversible vascular-neurodegenerative cascade initiation. PBS-based risk categorization could support shared decision-making regarding aggressive vascular risk management, lifestyle modifications, and consideration of emerging disease-modifying therapies for those with high pathological burden. Regular neurological surveillance through annual cognitive assessments and MRI monitoring for subclinical vascular changes should be implemented for high-risk individuals. In research settings, PBS could enhance patient selection for clinical trials targeting mixed-pathology dementia and facilitate longitudinal monitoring of intervention efficacy. As biomarker and imaging technologies mature, adaptation of PBS into non-invasive clinical assessments may help translate neuropathological insights into practical precision medicine approaches for dementia prevention and treatment.

### 4.6 Limitations

Several limitations should be acknowledged in interpreting these findings. The cross-sectional design limits our ability to establish temporal relationships between pathological processes and their clinical consequences. The retrospective nature of data from the NACC database introduces potential biases that may limit generalizability. The study population represents participants from specialized Alzheimer's Disease Research Centers, who may systematically differ from the general population in socioeconomic status, education levels, healthcare access, and research participation willingness. NACC participants may also represent a selected group with heightened awareness of cognitive symptoms or family history, introducing referral bias that could inflate observed pathological associations.

Survival bias represents a significant limitation, particularly in older age groups and autopsy-based studies. Individuals with severe pathological burden and aggressive disease trajectories may experience higher mortality before autopsy, leading to systematic underrepresentation of severely affected cases. This selective survival could explain: (1) apparent attenuation of pathological burden effects in older participants; (2) counterintuitive findings such as unexpectedly lower AD risk (24.1 %) in non-stroke participants with very high pathological burden, potentially reflecting preferential survival of resilient individuals; and (3) underestimation of true pathological burden effects in the general population. This bias may be particularly pronounced in stroke-stratified analyses, where individuals face competing mortality risks.

Additionally, retrospective reliance on autopsy data creates selection biases, as brain donors may differ systematically from non-donors. The age-dependent patterns observed might partially reflect differential survival patterns rather than purely biological effects. The PBS, while comprehensive, was constructed using available variables and may not capture all relevant pathological processes. Future longitudinal studies with systematic mortality tracking, competing risk analyses, and diverse population sampling would be essential to enhance generalizability and disentangle biological effects from survival bias artifacts.

### 4.7 Future directions

Future research should refine PBS using machine learning to determine optimal pathological domain weights beyond equal weighting assumptions. Integrating additional markers such as TDP-43 pathology, α-synuclein deposits, and microglial activation may enhance predictive accuracy. Longitudinal studies are needed to track pathological burden accumulation and assess relationships with cognitive decline trajectories.

Investigating genetic factors moderating pathological burden-clinical relationships, particularly APOE genotype and vascular variants, could provide insights into individual vulnerability. Developing risk models integrating genetic, pathological, and clinical factors will support precision medicine. Biomarker development should focus on neuroimaging and fluid correlates reflecting neuropathological burden.

Implementation studies should develop simplified PBS versions, establish risk stratification thresholds, and design clinical decision support tools. Validation across diverse populations is essential for generalizability, while evaluating cost-effectiveness of PBS-guided interventions will inform clinical adoption.

## 5 Conclusions

This study demonstrates that cumulative pathological burden significantly determines AD risk, with important interactions involving stroke history and age. Comprehensive pathological burden assessment tools could enhance risk stratification and guide therapeutic strategies. The pronounced age-dependent effects underscore the need for age-specific prevention approaches, particularly aggressive vascular risk management in younger populations. These findings advance understanding of AD pathophysiology and support personalized dementia care strategies.

## Ethics approval and consent to participate

Not applicable. All data were downloaded from the internet.

## Human ethics and consent to participate declarations

Not applicable.

## Participate declaration

Not applicable.

## Consent for publication

Not applicable.

## Availability of data and materials

The data used in the present study are all publicly available at https://naccdata.org/requesting-data/nacc-data.

## Funding

The author(s) declare financial support was received for the research, authorship, and/or publication of this article. This work was supported by the Science and Technology Program of Fujian Provincial Health Commision, China (Grant No.2023TG01010056)

## CRediT authorship contribution statement

**Fen Liu:** Writing – review & editing, Writing – original draft. **Xuesong Xia:** Writing – original draft. **Chengjie Zheng:** Writing – review & editing. **Feng Liu:** Writing – review & editing. **Min Jiang:** Writing – review & editing.

## Declaration of competing interest

The authors declare that they have no known competing financial interests or personal relationships that could have appeared to influence the work reported in this paper.

## Appendix Supplementary materials

Image, application 1
