# Corrigendum to Synergistic Effects of Multiple Pathological Processes on Alzheimer's Disease Risk: Evidence for Age-Dependent Stroke Interactions [The Journal of Prevention of Alzheimer's Disease (2025) 100268]

**DOI:** 10.1016/j.tjpad.2025.100371

**Published:** 2025-09-25

**Authors:** Fen Liu, Xuesong Xia, Chengjie Zheng, Feng Liu, Min Jiang

**Affiliations:** a Department of Neurology, Shengli Clinical Medical College of Fujian Medical University, Fujian Provincial Hospital, Fuzhou University Affiliated Provincial Hospital, No.134, Dong Street, Fuzhou 350001, Fujian Province, China; b Department of Hepatobiliary Pancreatic Surgery, Shengli Clinical Medical College of Fujian Medical University, Fujian Provincial Hospital, Fuzhou University Affiliated Provincial Hospital, No.134, Dong Street, Fuzhou 350001, Fujian Province, China; c The First Affiliated Hospital, College of Clinical Medicine of Henan University of Science and Technology, Luoyang 471000, Henan Province, China; d Department of Anesthesiology, Shengli Clinical Medical College of Fujian Medical University, Fujian Provincial Hospital, Fuzhou University Affiliated Provincial Hospital, No.134, Dong Street, Fuzhou 350001, Fujian Province, China

The authors regret that the financial support statement should be corrected as follows: The author(s) declare financial support was received for the research, authorship, and/or publication of this article. Sponsored by 10.13039/501100017686Fujian provincial health technology project (2023TG001).

The authors would like to apologise for any inconvenience caused.

