# Supplementary material for: Synergistic effects of multiple pathological processes on Alzheimer's disease risk: Evidence for age-dependent stroke interactions
Source: J Prev Alzheimers Dis. 2025 Jul 15;12(8):100268. doi: 10.1016/j.tjpad.2025.100268 (PMC12413705; doi:10.1016/j.tjpad.2025.100268)
Supplement: Supplementary file 1 [file mmc1.docx]

Supplementary materials: Table1. Neuropsychological Test Performance by Diagnostic Group

| Characteristics | AD(N=6003) | Control(N=4920) | AD + Stroke(N=385) | Total(N=11308) |
| --- | --- | --- | --- | --- |
| MMSE |  |  |  |  |
| Mean±SD | 21.65±6.49 | 28.90±1.29 | 22.17±6.22 | 24.82±6.10 |
| Median[min-max] | 23.00[0.0e+0,30.00] | 29.00[18.00,30.00] | 24.00[0.0e+0,30.00] | 27.00[0.0e+0,30.00] |
| BOSTON |  |  |  |  |
| Mean±SD | 26.83±20.55 | 28.25±7.43 | 28.36±22.31 | 27.50±16.30 |
| Median[min-max] | 24.00[0.0e+0,98.00] | 28.00[0.0e+0,98.00] | 25.00[0.0e+0,98.00] | 27.00[0.0e+0,98.00] |
| LOGIMEM |  |  |  |  |
| Mean±SD | 11.82±23.64 | 15.40±8.57 | 14.98±26.64 | 13.48±18.86 |
| Median[min-max] | 5.00[0.0e+0,98.00] | 15.00[0.0e+0,98.00] | 6.00[0.0e+0,98.00] | 10.00[0.0e+0,98.00] |
| MEMUNITS |  |  |  |  |
| Mean±SD | 10.26±24.92 | 14.35±8.76 | 13.83±28.21 | 12.16±19.85 |
| Median[min-max] | 2.00[0.0e+0,98.00] | 14.00[0.0e+0,98.00] | 3.00[0.0e+0,98.00] | 8.00[0.0e+0,98.00] |
| DIGIF |  |  |  |  |
| Mean±SD | 12.23±20.82 | 9.35±8.82 | 14.31±24.45 | 11.05±16.93 |
| Median[min-max] | 7.00[0.0e+0,98.00] | 8.00[2.00,98.00] | 8.00[0.0e+0,98.00] | 8.00[0.0e+0,98.00] |
| DIGIFLEN |  |  |  |  |
| Mean±SD | 11.16±21.09 | 7.55±8.85 | 13.23±24.71 | 9.66±17.16 |
| Median[min-max] | 6.00[0.0e+0,98.00] | 7.00[0.0e+0,98.00] | 6.00[0.0e+0,98.00] | 6.00[0.0e+0,98.00] |
| DIGIB |  |  |  |  |
| Mean±SD | 10.71±22.54 | 7.70±9.18 | 12.30±25.47 | 9.46±18.19 |
| Median[min-max] | 5.00[0.0e+0,98.00] | 7.00[0.0e+0,98.00] | 5.00[0.0e+0,98.00] | 6.00[0.0e+0,98.00] |
| DIGIBLEN |  |  |  |  |
| Mean±SD | 9.72±22.74 | 5.83±9.22 | 11.41±25.69 | 8.08±18.38 |
| Median[min-max] | 4.00[0.0e+0,98.00] | 5.00[0.0e+0,98.00] | 4.00[0.0e+0,98.00] | 4.00[0.0e+0,98.00] |
| ANIMALS |  |  |  |  |
| Mean±SD | 16.39±20.02 | 20.03±8.52 | 18.11±23.52 | 18.03±16.32 |
| Median[min-max] | 12.00[0.0e+0,98.00] | 19.00[1.00,98.00] | 12.00[0.0e+0,98.00] | 15.00[0.0e+0,98.00] |
| VEG |  |  |  |  |
| Mean±SD | 13.17±22.24 | 14.65±9.63 | 14.67±24.57 | 13.86±18.00 |
| Median[min-max] | 8.00[0.0e+0,98.00] | 14.00[1.00,98.00] | 8.00[0.0e+0,98.00] | 11.00[0.0e+0,98.00] |
| TRAILA |  |  |  |  |
| Mean±SD | 175.74±305.81 | 57.50±139.38 | 198.09±331.30 | 125.06±255.64 |
| Median[min-max] | 54.00[12.00,998.00] | 34.00[4.00,998.00] | 58.00[16.00,998.00] | 42.00[4.00,998.00] |

*Note: Data presented as mean±SD with median and range. Higher scores indicate better performance except for Trail Making Test A where lower scores reflect better performance. Scores of 95-98 represent various inability-to-test codes which may inflate means in impaired groups. MMSE = Mini-Mental State Examination; BOSTON = Boston Naming Test; LOGIMEM = Logical Memory; MEMUNITS = Memory Units; DIGIF/DIGIB = Digit Span Forward/Backward; DIGIFLEN/DIGIBLEN = Digit Span Forward/Backward Length; ANIMALS/VEG = Animal/Vegetable Fluency; TRAILA = Trail Making Test A.*

Supplementary materials: Table2. Neuropathological Features by Diagnostic Group

| Characteristics | AD(N=6003) | Control(N=4920) | AD + Stroke(N=385) | Total(N=11308) | pvalue | FDR |
| --- | --- | --- | --- | --- | --- | --- |
| Braak |  |  |  |  | 0.00E+00 | 0.00E+00 |
| 0 | 94(0.83%) | 124(1.10%) | 1(8.8e-3%) | 219(1.94%) |  |  |
| B1 | 484(4.28%) | 1385(12.25%) | 33(0.29%) | 1902(16.82%) |  |  |
| B2 | 955(8.45%) | 2281(20.17%) | 117(1.03%) | 3353(29.65%) |  |  |
| B3 | 4470(39.53%) | 1130(9.99%) | 234(2.07%) | 5834(51.59%) |  |  |
| CERAD |  |  |  |  | 0.00E+00 | 0.00E+00 |
| C0 | 679(6.00%) | 1961(17.34%) | 62(0.55%) | 2702(23.89%) |  |  |
| C1 | 456(4.03%) | 1083(9.58%) | 38(0.34%) | 1577(13.95%) |  |  |
| C2 | 1193(10.55%) | 894(7.91%) | 112(0.99%) | 2199(19.45%) |  |  |
| C3 | 3675(32.50%) | 982(8.68%) | 173(1.53%) | 4830(42.71%) |  |  |
| Thal |  |  |  |  | 0.00E+00 | 0.00E+00 |
| A0 | 334(2.95%) | 828(7.32%) | 42(0.37%) | 1204(10.65%) |  |  |
| A1 | 455(4.02%) | 1040(9.20%) | 30(0.27%) | 1525(13.49%) |  |  |
| A2 | 491(4.34%) | 953(8.43%) | 38(0.34%) | 1482(13.11%) |  |  |
| A3 | 4723(41.77%) | 2099(18.56%) | 275(2.43%) | 7097(62.76%) |  |  |
| White Matter Rarefaction |  |  |  |  | 1.60E-08 | 1.60E-08 |
| Mild | 1844(16.31%) | 1638(14.49%) | 140(1.24%) | 3622(32.03%) |  |  |
| Moderate | 1331(11.77%) | 983(8.69%) | 93(0.82%) | 2407(21.29%) |  |  |
| None | 2210(19.54%) | 1927(17.04%) | 117(1.03%) | 4254(37.62%) |  |  |
| Severe | 618(5.47%) | 372(3.29%) | 35(0.31%) | 1025(9.06%) |  |  |
| Brain Weight |  |  |  |  |  |  |
| Mean±SD | 1141.69±157.18 | 1203.70±139.97 | 1153.30±122.43 | 1169.06±151.90 |  |  |
| Median[min-max] | 1140.00[560.00,1950.00] | 1196.00[801.00,1643.00] | 1138.00[890.00,1500.00] | 1160.00[560.00,1950.00] |  |  |

*Note: Data presented as n (%) for categorical variables and mean±SD for continuous variables. Braak stages: 0 = no tangles, B1-B3 = increasing tangle burden. CERAD: C0-C3 = increasing neuritic plaque density. Thal phases: A0-A3 = increasing amyloid-β deposition extent.*
